# Assessment of Hyaluronic Acid Filler in Gluteal Augmentation and Contouring: A 1‐Year Prospective Study

**DOI:** 10.1111/jocd.70600

**Published:** 2025-12-21

**Authors:** Maria Bussade, Gladstone Faria, Marta Barros, Caroline Mourão, Melina Kichler, Renata Viana, Luciana Zattar, Marcelle Almeida de Sousa Nogueira, Ricardo Boggio

**Affiliations:** ^1^ MD Institute São Paulo Brazil; ^2^ Instituto Boggio São Paulo Brazil; ^3^ Independent Investigator São Paulo Brazil; ^4^ University of São Paulo (USP) São Paulo Brazil

**Keywords:** body contouring, dermal filler, gluteal augmentation, hyaluronic acid, patient satisfaction

## Abstract

**Background:**

Minimally invasive gluteal augmentation techniques have gained popularity as alternatives to surgical procedures, offering reduced downtime and an improved safety profile. Hyaluronic acid (HA) fillers have emerged as a promising option due to their biocompatibility and favorable rheological characteristics.

**Objectives:**

To evaluate safety, efficacy, durability, and patient satisfaction of a cross‐linked HA filler for gluteal augmentation and contouring over a 12‐month period, and to conduct a pilot exploratory substudy assessing serum HA levels up to 90 days.

**Methods:**

This was a 12‐month, prospective, multicenter, open‐label study including 30 healthy women undergoing gluteal augmentation with a cross‐linked HA filler (UP Max). Each participant received up to 60 mL of HA filler, administered using a quadrant‐based technique, guided by anatomical landmarks. Outcomes included safety (adverse events), efficacy (standardized photography and 3D imaging, ultrasound), durability and patient satisfaction. In addition, serum HA levels were measured in a pilot subgroup of patients.

**Results:**

The mean injected volume was 43.60 ± 11.48 mL. Patient satisfaction peaked at Day 30 (90%) and declined to 62% at 12 months, consistent with HA biodegradation. Ultrasound confirmed subcutaneous placement and progressive filler absorption and integration, with no signs of migration, fibrosis or nodularity. Serum HA levels showed a transient increase at Day 30 and returned to near baseline at Day 90. No serious adverse events were reported, and minor complications were infrequent.

**Conclusions:**

HA filler demonstrated a favorable safety profile, high patient satisfaction, and aesthetic improvements over 1 year. These findings support its use as a safe and effective option for nonsurgical gluteal augmentation and body contouring.

## 1 Introduction

The demand for gluteal augmentation procedures has increased significantly, with a 60.9% increase between 2019 and 2023, according to The International Society of Aesthetic Plastic Surgery [1]. Historically, surgical techniques such as implant‐based augmentation and autologous fat grafting have been the predominant approaches. However, these methods are associated with extended recovery periods and surgical risks, leading to a growing interest in less invasive approaches. In this context, hyaluronic acid (HA)‐based fillers have emerged as a transformative option, offering predictable and natural‐looking outcomes with minimal downtime [2, 3, 4].

HA fillers have gained traction due to their biocompatibility, biodegradability, and ability to integrate into subcutaneous tissues [5], making them suitable for restoring volume and refining body contours [6, 7, 8]. Their rheological characteristics enable effective volumization, projection, and structural support in high‐pressure anatomical areas [9, 10, 11]. These properties have supported their increasing use in gluteal augmentation, with studies demonstrating immediate aesthetic improvements and consistent outcomes across diverse patient profiles [2, 3, 4, 12]. Clinical evidence consistently demonstrates the longevity of HA gels lasting up to 20–24 months, while achieving and maintaining high patient satisfaction rates across multiple studies [3, 12, 13].

This study aimed to evaluate the safety, efficacy, durability and patient satisfaction of a cross‐linked HA gel filler for gluteal augmentation and harmonization, contributing to the ongoing advancement in the field of minimally invasive body contouring techniques over a 12‐month period. In addition, a pilot exploratory substudy assessed serum HA levels up to 90 days to provide preliminary insights into systemic kinetics after large‐volume filler injection.

## 2 Materials and Methods

### 2.1 Study Design

This was a 12‐month prospective, multicenter, open‐label study conducted at two clinical centers in Brazil. The study aimed to generate clinical evidence on the performance of a cross‐linked hyaluronic acid‐based filler on gluteal augmentation, with structured assessments at baseline (Day 0), and at Days 30, 90, 180, and 360 post‐procedure.

The primary objective was to assess the product's safety and efficacy in gluteal augmentation, while secondary endpoints included patient satisfaction and durability. In addition to the main clinical assessments, a pilot exploratory substudy evaluated serum hyaluronic acid (sHA) levels measured using a quantitative ELISA kit.

### 2.2 Participants

The main study inclusion criteria were women aged 20–45 years willing to undergo gluteal augmentation. Exclusion criteria included a history of keloid or hypertrophic scar formation, known allergies to lidocaine or hyaluronic acid, and menopausal status. Additional exclusions were chronic diseases, underweight or overweight participants (body mass index [BMI] < 18.5 or ≥ 30 kg/m^2^), or significant weight fluctuations within the past 6 months.

### 2.3 Product

The dermal filler used in this study, UP Max (Ilikia—CGBio Co. Ltd., South Korea), is a cross‐linked hyaluronic acid‐based gel. Each syringe contains 3 mL of HA (20 mg/mL) with 0.3% lidocaine. The gel features a multiphasic structure developed using proprietary Revolution‐Rotation (*R* ^2^) technology, which enables uniform distribution of cross‐linked HA gel around fully hydrated, cross‐linked macroparticles [14, 15]. The product is registered in Brazil for soft tissue augmentation but not specifically for the gluteal region; therefore, this indication is considered off‐label. Nevertheless, its rheological properties make it suitable for the volumization of large anatomical areas [10].

### 2.4 Procedure

#### 2.4.1 Marking

**FIGURE 1:**
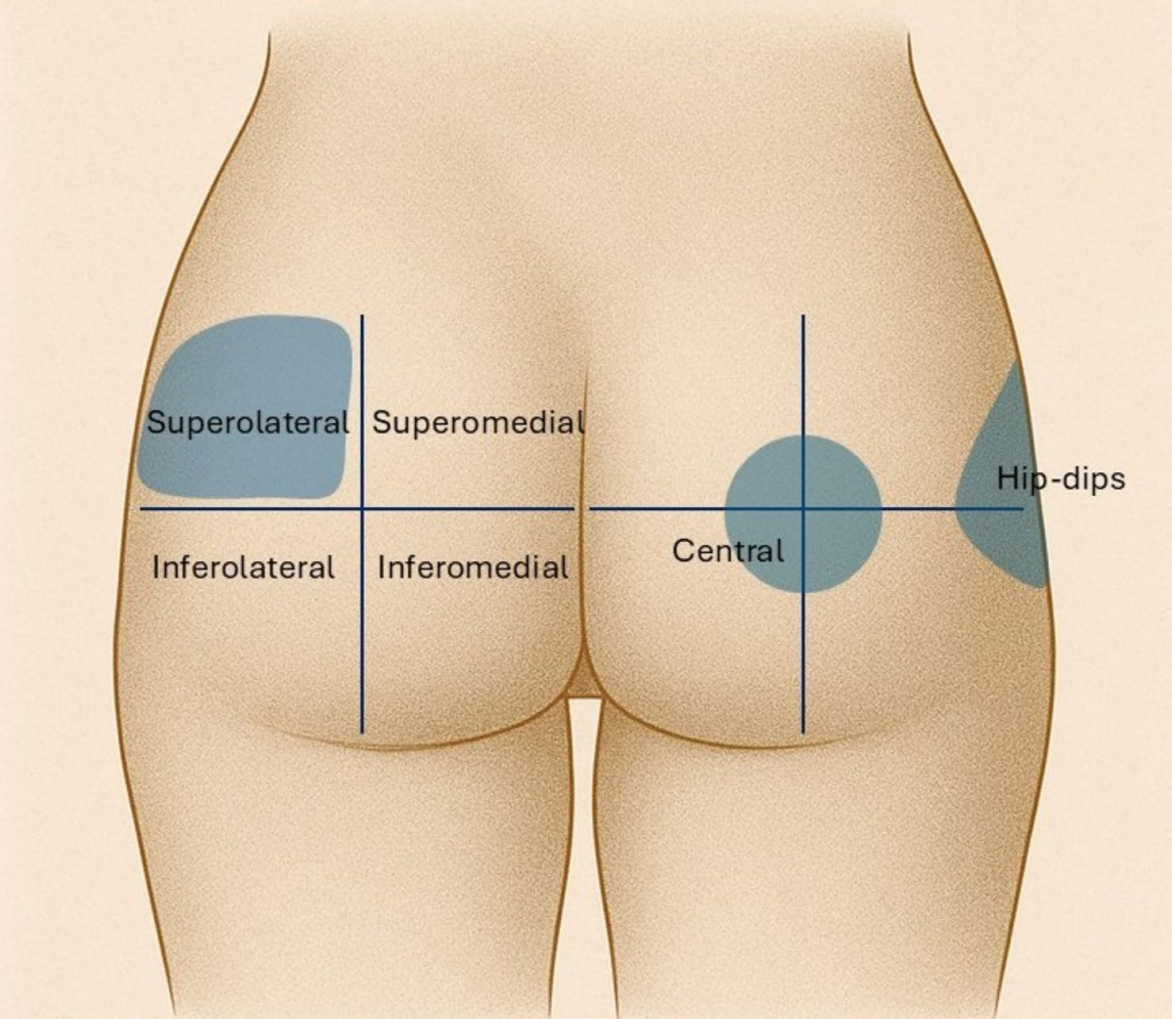
Anatomical division of the gluteal area into quadrants used for HA filler planning. Main injection areas are highlighted, including the hip dips area when indicated.

The planning process is essential to ensure symmetrical distribution of the filler within the gluteal region. Marking was performed with the patient in a standing position to allow accurate anatomical visualization. Each buttock was divided into four quadrants using defined anatomical landmarks. The vertical axis is based on the midline of the thighs, dividing the buttock into medial (inner) and lateral (outer) halves. A horizontal axis is drawn at the level of the greater trochanter, separating the superior (upper) and inferior (lower) gluteal zones. The intersection of these axes defines four quadrants – superolateral, superomedial, inferolateral, and inferomedial– with the central intersection representing the core injection zone. In patients presenting trochanteric depression (“*hip dips*”), the area was also marked and addressed when necessary (Figure 1).

#### 2.4.2 Injection Procedure

As illustrated in Figure 1, the injection technique was planned with the patient in a standing position, following a structured anatomical marking strategy and individualized according to each patient's anatomical features and aesthetic goals. The maximum total volume administered per patient was 60 mL. All patients received an initial filler deposition in the superolateral quadrant, which was prioritized due to its critical role in enhancing upper pole projection and defining the gluteal contour. Subsequent filler distribution was tailored to the patient's individual anatomy and needs, considering baseline contour, tissue laxity, and desired enhancement, with additional injections in the superomedial, inferolateral, and central zones, to optimize volumetric balance, symmetry, and natural‐looking contour. Hip‐dips deformities were also addressed when indicated further contributing to improved gluteal harmony and aesthetic proportionality.

All procedures were performed under aseptic conditions with patients in the prone position to allow optimal access and symmetrical product distribution. Local anesthesia with 2% lidocaine was administered at the entry point. The main entry point was located at the intersection of the medial and lateral marking lines, providing direct access to the superolateral quadrant, which is the primary area for volumization and contour enhancement. Additional entry points were used as needed, depending on gluteal size and individual anatomy, particularly to reach the trochanteric depression (hip dip) area when this region was treated.

The filler was delivered using an 18G × 70 mm blunt‐tipped cannula via retrograde linear injection technique, ensuring uniform product deposition. Strict subcutaneous plane placement must be maintained, approximately 0.5–1 cm beneath the skin and superficial to the superficial fascia, and adherence to anatomical safety guidelines to minimize the risk of intravascular or intramuscular deposition.

After injection, manual massage was applied to ensure even distribution, molding and proper filler accommodation. A sterile dressing was applied to the entry points and patients were advised to limit intense physical activity for at least 48 h.

### 2.5 Outcomes

#### 2.5.1 Photographic Evaluation

Standardized 2D and 3D photographs were obtained at all assessment points to assess volumetric changes and contour improvements. 3D images were captured using the LifeViz system (QuantifiCare, France), which generated 360° high‐resolution images.

#### 2.5.2 Patient Satisfaction Assessment

Patient‐reported outcomes were assessed using the Global Aesthetic Improvement Scale (GAIS), which rated their perceived aesthetic satisfaction as “very much improved”, “much improved”, “improved”, “no change”, or “worse” [16] compared to baseline, administered at each follow‐up visit to capture overall treatment satisfaction.

#### 2.5.3 Ultrasound Analysis

High‐frequency ultrasound was employed as a noninvasive imaging modality to support anatomical evaluation before and after the procedure. The objectives were to confirm correct filler placement within the subcutaneous plane, to monitor tissue degradation and integration over time, and to assess local tissue response throughout follow‐up. Two systems were used for image acquisition: the *Canon Viamo C100* (Canon Medical Systems, Japan), with an 18 MHz linear probe, and the *GE Venue Fit* (GE Healthcare, USA), with a 20 MHz probe. Qualitative analysis included the evaluation of the filler distribution and the surrounding tissue for signs of edema, fibrosis, or inflammatory response. All examination parameters were performed in both longitudinal and transverse planes to ensure accuracy and reproducibility, and all scans were performed and interpreted by a single experienced physician across both centers.

#### 2.5.4 Serum Hyaluronic Acid Analysis

Venous blood was collected at baseline (pre‐procedure), 24 h, and at Days 7, 30, and 90. The analysis was conducted as an exploratory pilot sub‐study including three patients. sHA concentrations were measured using a hyaluronan enzyme‐linked immunosorbent assay (HA‐ELISA) kit (Echelon Biosciences Inc., Cat. No. K‐1200), according to the manufacturer's protocol. A 100 μL of each serum sample or HA standard was incubated with 50 μL of HA detector solution in a 96‐well incubation plate at 37°C for 1 h. Subsequently, 100 μL of this mixture was transferred to a detection plate pre‐coated with HA, followed by a 30‐min incubation at 4°C. After washing, 100 μL of diluted enzyme solution was added to each well and incubated at 37°C for 30 min. Following a second wash step, 100 μL of a working substrate solution (*p*‐nitrophenyl phosphate) was added and incubated for 30 min at room temperature in the dark. The reaction was stopped with 50 μL of stop solution, and absorbance was read at 405 nm using a microplate reader. All samples were analyzed in duplicate. HA concentrations were calculated by interpolation from a standard curve generated using known HA standards (0 to 1600 ng/mL), applying a four‐parameter logistic (4PL) regression model. Results were expressed in nanograms per milliliter (ng/mL).

## 3 Results

### 3.1 Demographics and Procedural Information

This study included 30 female participants, with a mean age of 34.27 ± 7.95 years (range: 23–53) and a mean BMI of 21.80 ± 2.39 kg/m^2^. Four participants (13%) had previously received biostimulator treatments in the gluteal area (at least 1 year prior to study enrollment). All participants were in good health, without a history of chronic conditions or contraindications to the procedure (Table 1).

**TABLE 1:** Demographic characteristics of the study participants.

Attribute	Mean ± SD
Age	34.27 ± 7.95
Body mass index (BMI)	21.80 ± 2.39
Fitzpatrick phototype	*n*
I	1
II	7
III	9
IV	10
V	3
VI	0
Gluteal prior procedures	4

The mean volume of HA filler injected per patient was 43.60 ± 11.48 mL, ranging from 18 to 60 mL, with the superolateral quadrant prioritized as the initial injection site. Subsequent filler distribution was adapted according to individual anatomy and aesthetic goals, including treatment of hip dips when indicated.

Procedure‐related pain was assessed immediately post‐procedure using a 0–10 numeric rating scale. The mean score was 1.74 ± 1.76, indicating the procedure was well tolerated and associated with only mild discomfort.

### 3.2 Standardized Photographic Follow‐Up

**FIGURE 2:**
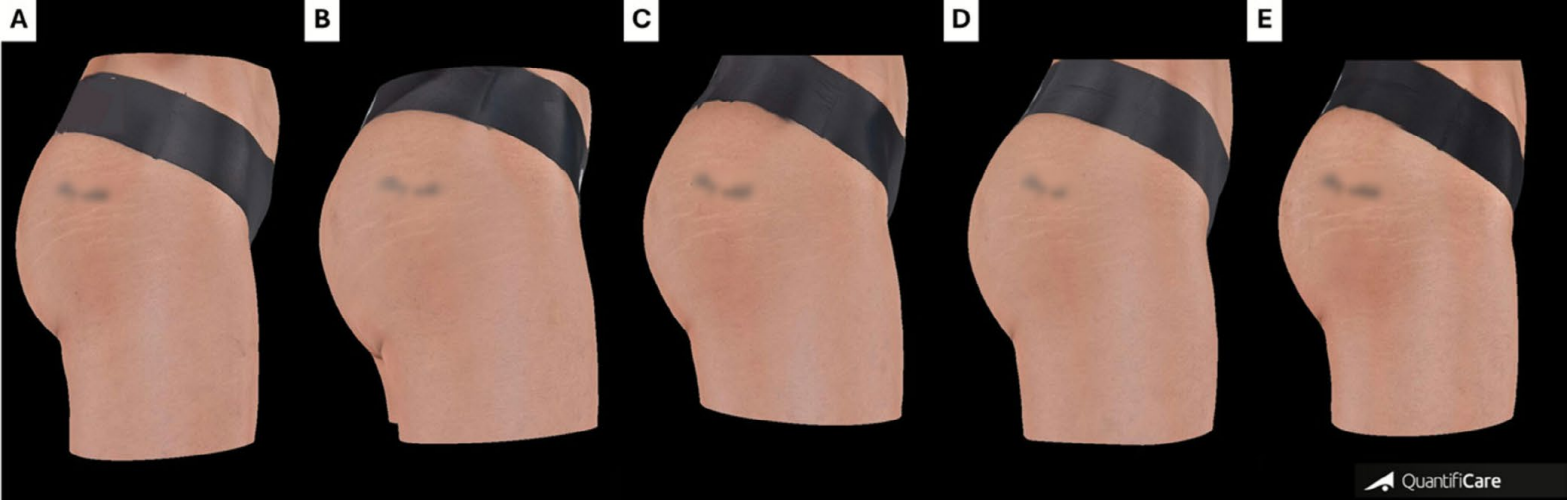
Standardized 3D imaging follow‐up of a patient treated with 18 mL of HA filler: (A) pre‐procedure; (B) 30 days; (C) 90 days; (D) 180 days; and (E) 360 days post‐procedure.

**FIGURE 3:**
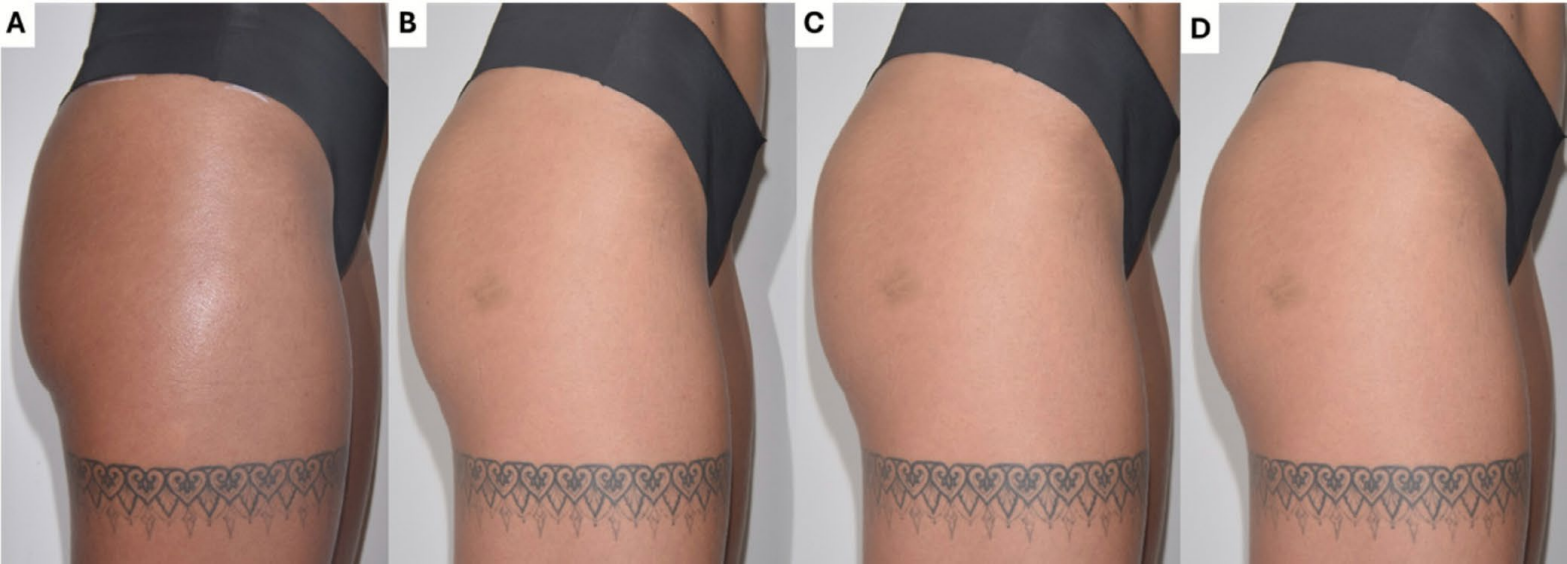
Standardized 3D imaging follow‐up of a patient treated with 60 mL of HA filler: (A) pre‐procedure; (B) 30 days; (C) 90 days; and (D) 180 days post‐procedure.

**FIGURE 4:**
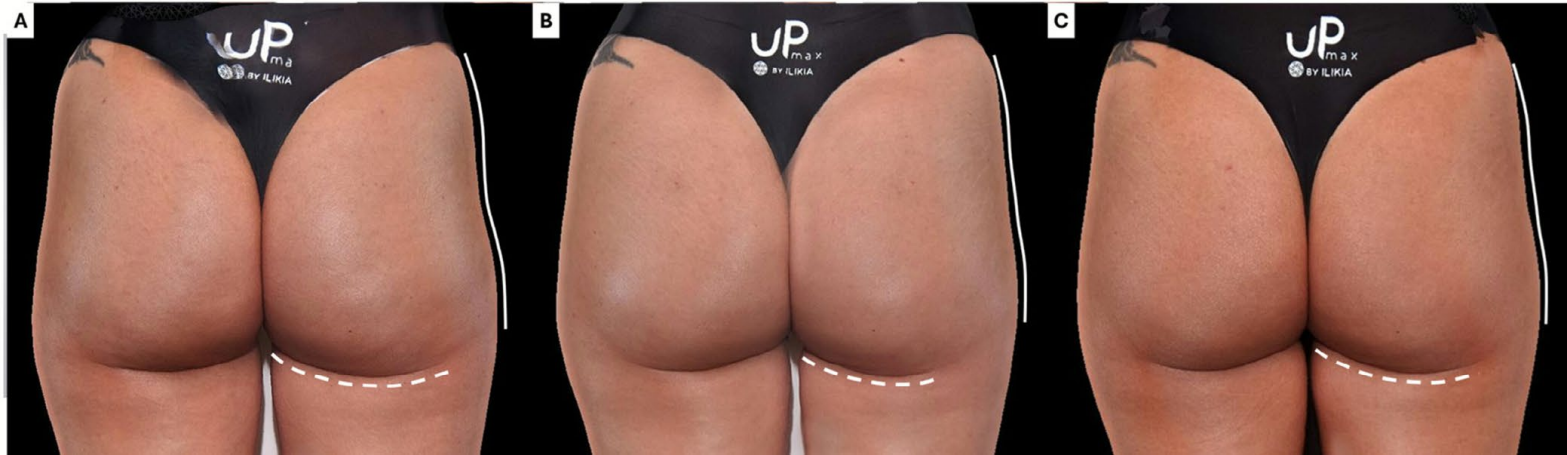
3D imaging follow‐up of a patient treated with 60 mL of HA filler: (A) baseline; (B) 30 days post‐procedure; and (C) 360 days post‐procedure. Trochanteric depressions (hip dips) and infragluteal fold are highlighted to illustrate anatomical changes.

Standardized photographic documentation was obtained at baseline and all follow‐up points to assess volumetric changes, contour improvements, and overall aesthetic outcomes (Figures 2 and 3). Improvement in trochanteric depressions (“hip dips”)—a common concern among patients—was observed (Figure 4). Additionally, a visible reduction in the infragluteal fold was documented, reflecting a lifting effect achieved in the treated area.

### 3.3 Patient Satisfaction

**FIGURE 5:**
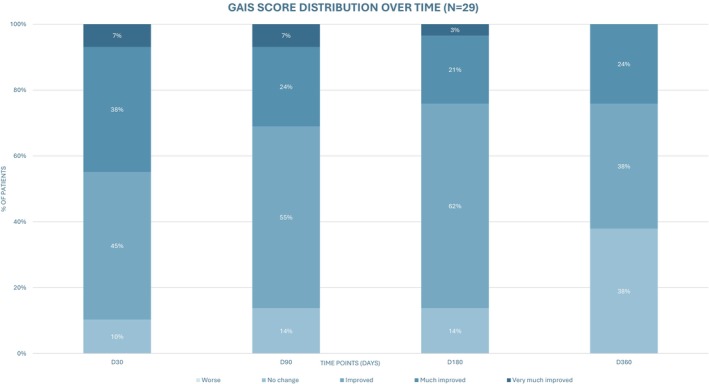
Distribution of Global Aesthetic Improvement Scale (GAIS) scores over time among the 29 patients who completed the follow‐up visits. The bar graph shows the proportion of responses and the absolute number of patients (*n*) for each GAIS category at Days 30, 90, 180, and 360 post‐treatment.

Of the 30 enrolled participants, 29 completed all follow‐up visits and were included in the GAIS analysis. One participant became pregnant during the study period and was excluded from this evaluation due to body weight fluctuations. At Day 30, 26 patients (90%) reported improvement, primarily classified as “improved” or “much improved”. By Day 180, 25 (86%) still perceived improvement—“improved,” “much improved” and “very much improved”. At Day 360, 18 (62%) continued to report some degree of aesthetic improvement. Notably, none reported worsening at any follow‐up point. These results are illustrated in Figure 5.

### 3.4 Serum Hyaluronic Acid Levels

**FIGURE 6:**
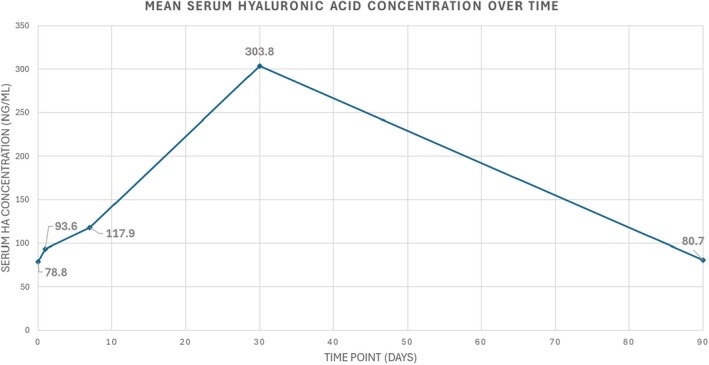
Mean serum hyaluronic acid (sHA) concentration over time (*n* = 3). Samples were collected at baseline, 24 h post‐procedure, and at Days 7, 30 and 90 post‐injection using ELISA. Connecting lines are used for visualization and do not indicate continuous interpolation.

In a subset of participants (*n* = 3), sHA levels were measured at baseline (pre‐procedure), 24 h, and at Days 7, 30, and 90 post‐injection using ELISA. Mean sHA concentrations increased from 78.8 ng/mL at baseline to 117.9 ng/mL at Day 7, peaking at 303.8 ng/mL at Day 30. By Day 90, levels declined to 80.7 ng/mL, returning close to baseline values (Figure 6). It is important to note that serum HA was assessed at predetermined time points (baseline, 24 h, Days 7, 30, and 90), rather than continuously throughout the follow‐up period, due to ethical and logistical constraints on blood sampling frequency.

### 3.5 Ultrasound Results

**FIGURE 7:**
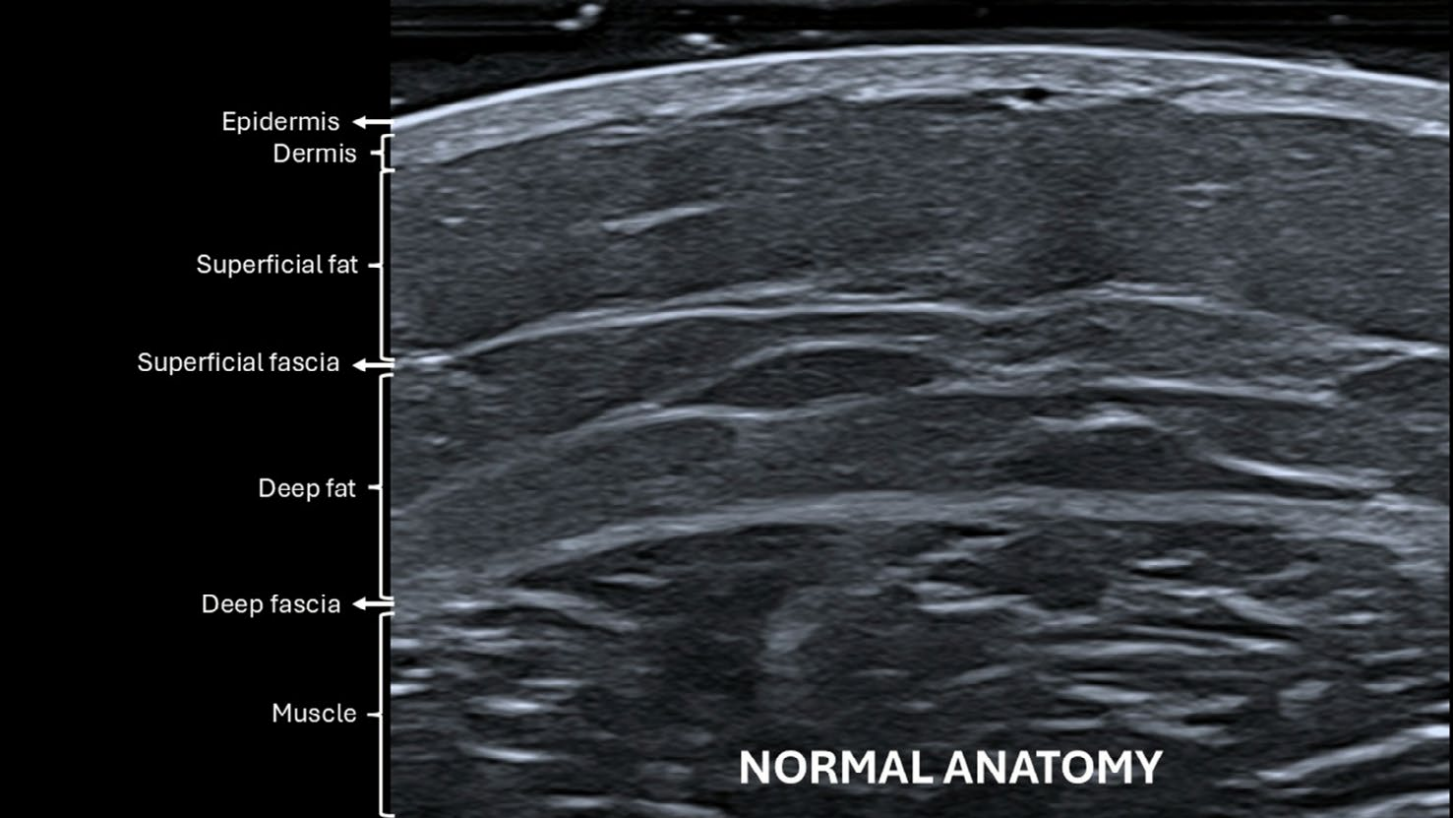
B‐mode ultrasound image (20 MHz) showing normal gluteal anatomy prior to treatment.

High‐frequency ultrasound assessment was performed at baseline to evaluate the gluteal region and identify any conditions that might contraindicate treatment. Normal gluteal anatomy scanned at baseline is illustrated in Figure 7. Ultrasound was also performed post‐procedure to confirm the subcutaneous plane of injection, and at the follow‐up visits to monitor product behavior, and detect potential adverse reactions over time.

**FIGURE 8:**
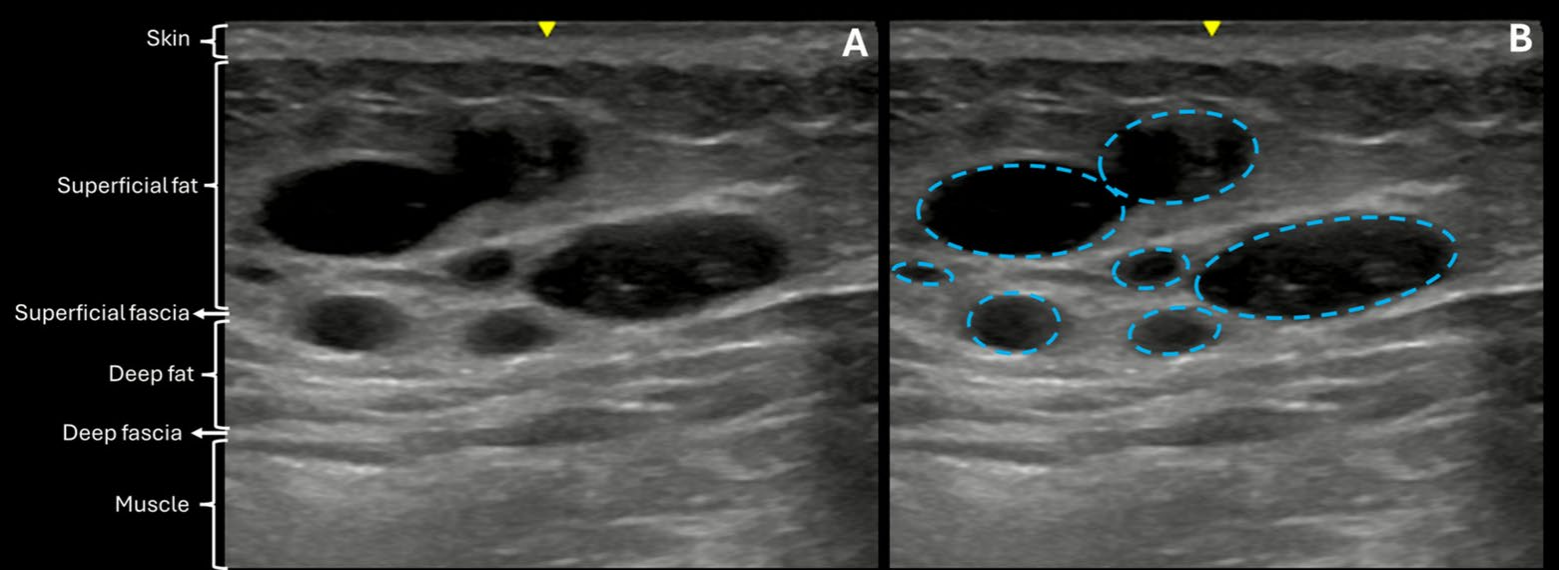
(A) US images (B‐Mode, 20 MHz) on Day 0. (B) The hyaluronic acid filler (blue dashed line) appears as a predominantly anechoic area in the subcutaneous plane.

On Day 0, immediately after injection, the product appeared as a well‐defined anechoic structure within the subcutaneous plane (Figure 8), consistent with its gel‐like consistency and differentiated from surrounding tissues.

**FIGURE 9:**
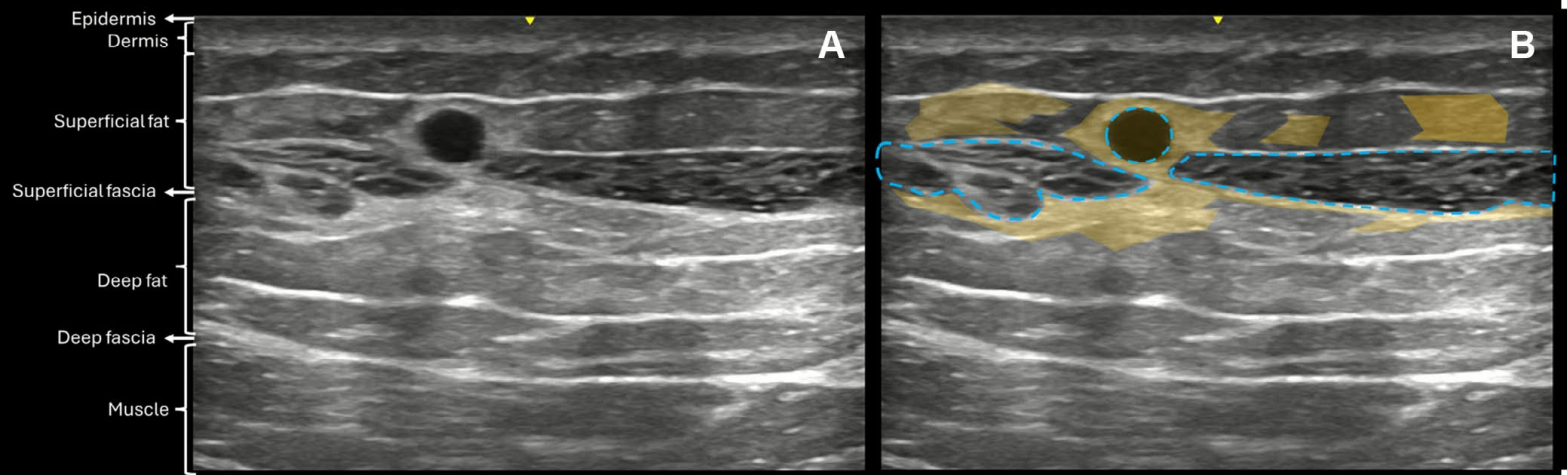
(A) US image (B‐Mode, 20 MHz) at Day 30 showing the hyaluronic acid filler in the subcutaneous plane. (B) Same image with overlays: filler marked by a blue dashed line and surrounding hyperechoic areas (yellow) indicating signs of tissue reaction.

At Day 30, early signs of surrounding tissue response were observed, characterized by hyperechoic changes in the adjacent subcutaneous tissue (Figure 9).

**FIGURE 10:**
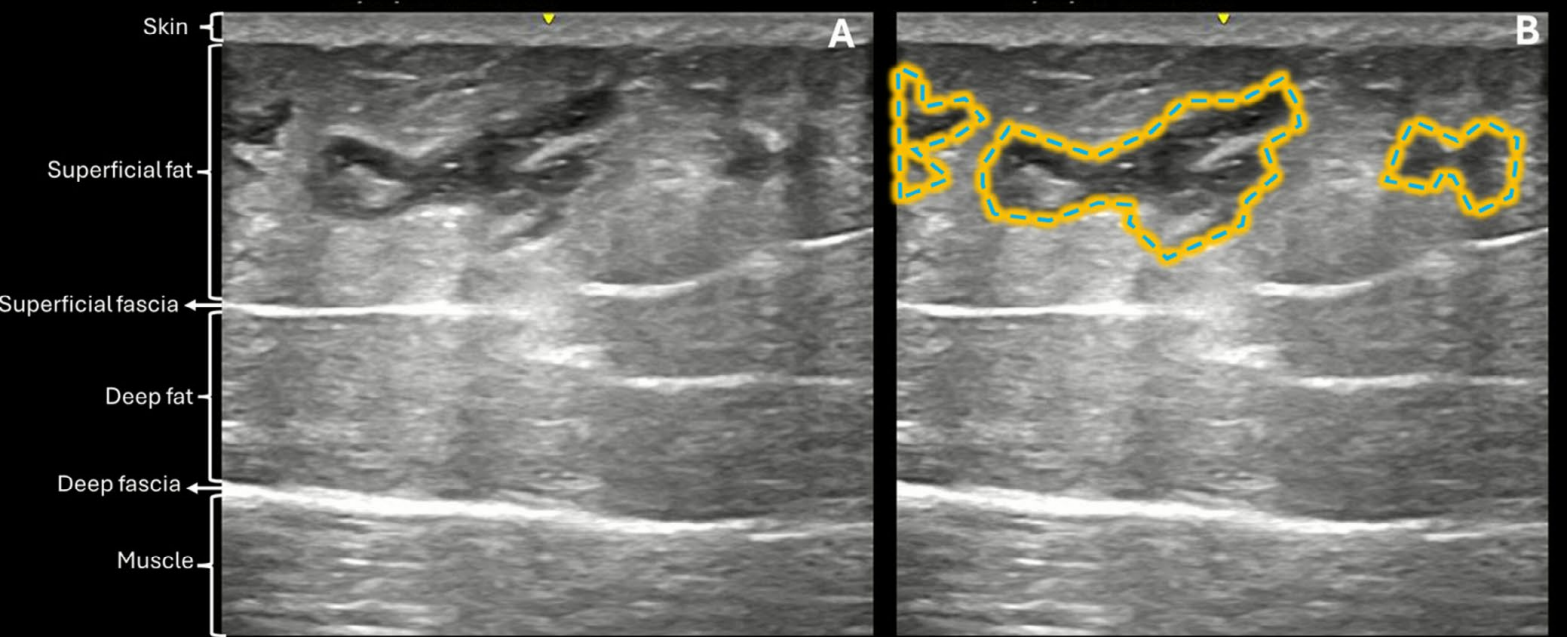
(A) US image (B‐mode, 20 MHz) of the gluteal region at Day 360. (B) Same image with overlays: the hyaluronic acid filler, outlined by a dashed line, appears as a predominantly hypoechoic material in the subcutaneous tissue.

By Day 360, the filler exhibited a predominantly hypoechoic pattern, indicating progressive integration into adipose tissue and partial bioresorption, while residual product remained visible at the injection plane in most patients (Figure 10).

A gradual change in echogenicity—from initially anechoic to progressively hypoechoic—was consistently observed, reflecting tissue interaction and structural transformation. The filler remained visualized and confined to the subcutaneous plane, throughout the 12‐month period, with no evidence of migration, calcification, fibrosis, or granuloma formation.

### 3.6 Adverse Events

Participants were actively monitored for adverse events throughout the follow‐up period, including remote contact with those who missed in‐person visits, ensuring comprehensive safety data collection.

No serious adverse events (SAEs) occurred during the 12‐month follow‐up and a total of four adverse events were reported. One patient developed a localized infection on Day 8, presenting erythema, warmth, swelling, and mild tenderness at the injection site, which was fully resolved with oral antibiotics (Cephalexin). Two participants experienced delayed, intermittent, persistent and mild swelling at Days 180 and 360. Both episodes were self‐limiting and occurred in the context of unrelated systemic inflammatory conditions. At the 6‐month visit, one patient reported persistent discomfort in the hip dip region, particularly when lying on her side. Ultrasound confirmed focal product accumulation, without signs of inflammation, likely due to localized overfilling. The area was treated with 3000 IU of hyaluronidase, resulting in complete resolution of the symptom.

## 4 Discussion

The use of HA fillers in the gluteal region was initially described for therapeutic purposes, particularly in HIV‐associated lipoatrophy, where it proved effective in restoring volume and improving quality of life [17]. Since then, indications have expanded to aesthetic procedures. Recent studies have investigated HA fillers for the correction of localized depressions such as hip dips, gluteal asymmetries, and cellulite [18, 19]. In parallel, there has been growing interest in their use for gluteal volumization and aesthetic enhancement, aiming to improve projection, contour, and overall body harmony [2, 12, 13, 20, 21]. Beyond the gluteal region, HA fillers have also been applied to other large‐volume anatomical areas, including calves, pectorals, shoulders, and abdomen [22, 23, 24, 25].

A key consideration in this context is the rheological suitability of HA gels for high‐volume and mechanically stressed anatomical regions. The filler used in this study has been characterized as having a high elastic and complex modulus, with predominantly elastic behavior, supporting its suitability for areas subject to compression and shear forces [10].

In this present prospective study, we evaluated safety, clinical efficacy, and patient satisfaction associated with a cross‐linked HA filler for gluteal augmentation. Beyond standard clinical and photographic assessments, we incorporated ultrasound and biochemical analyses to provide a comprehensive understanding of product behavior, performance, its integration and interaction within subcutaneous tissues.

Efficacy was demonstrated by consistent gluteal volumization and contour improvement documented by standardized 2D and 3D imaging. The technique yielded reproducible results, with 3D reconstructions confirming preservation of contour over time.

Satisfaction rates have been reported in previous studies [26, 27]. In a retrospective multicenter study of 35 participants undergoing gluteal augmentation with HA, a 94% satisfaction rate was observed among both patients and physicians using BODY‐Q scores [2]. Patient satisfaction in this study paralleled these findings, with high aesthetic improvement by 6 months, and 62% maintaining this perception at 1 year. The observed decline is consistent with progressive volume resorption as HA biodegrades and underscores the importance of discussing with patients the expected absorption profile of HA fillers and the potential need for retreatment to maintain optimal outcomes and cost–benefit expectations. Nevertheless, imaging data indicate that results at 1 year did not fully return to baseline, suggesting that partial integration into subcutaneous tissues or remodeling effects may contribute to sustained clinical benefit despite resorption [2, 26].

Injection volumes varied according to patient anatomy, body weight, and individual aesthetic goals. This personalized approach reflects real‐world clinical practice, where applying a fixed volume to all patients could result in unsatisfactory outcomes. Despite different injection volumes, collective analysis was performed, as all procedures followed a standardized protocol and outcomes were evaluated through both subjective (GAIS) and objective measures (photography, 3D imaging, and ultrasound).

Ultrasound analysis provided valuable objective evidence of product behavior and safety. Across more than 120 ultrasonographic evaluations, the HA filler was consistently visualized in the subcutaneous plane, with no evidence of migration or signs of serious adverse events, reflecting procedural accuracy, efficacy, safety and preservation of surrounding tissue integrity over time. A previous study has also demonstrated the long‐term behavior of body HA fillers using ultrasound imaging [13].

No serious adverse events (SAEs) were recorded, and minor complications were infrequent, consistent with the cumulative evidence for gluteal augmentation with cross‐linked HA fillers [26, 27, 28]. These findings suggest a more favorable risk–benefit profile compared to surgical augmentation techniques [2, 3, 27, 29]. Unlike fat grafting or lipoinjection, which carry a documented risk of fat embolism, no vascular or embolic events have been reported following HA filler implantation in the gluteal region. To date, only one solitary case report has described a possible HA‐related pulmonary embolism [30, 31].

Vascular safety remains a major concern in gluteal augmentation procedures, particularly because the region harbors large‐caliber vessels such as the superior and inferior gluteal arteries and the perforating branches of the profunda femoris. To minimize intravascular risk, all injections were performed in the superficial subcutaneous plane using an 18 G blunt‐tipped cannula, with low, controlled pressure and continuous retrograde movement, strictly avoiding the medial danger triangle where major neurovascular structures converge. These precautions are consistent with current anatomical safety guidelines for large‐volume filler injections and likely contributed to the absence of vascular complications observed in this study.

One patient, although satisfied with the aesthetic outcome, requested partial filler removal due to persistent discomfort in the hip dip area. Ultrasound imaging revealed focal product accumulation without signs of inflammation, consistent with localized overfilling. This case highlights the importance of volume planning, particularly in sensitive zones, avoiding large bolus deposition and favoring retrograde fan‐shaped injections to ensure uniform distribution and reduce the risk of product accumulation.

An exploratory substudy evaluated sHA levels in three participants to provide complementary information on HA kinetics after large‐volume filler injection in humans and systemic clearance. Baseline sHA was within the expected physiological range reported for healthy adults [11, 32]. A transient peak was observed at Day 30, with concentrations returning close to baseline by Day 90. All participants remained clinically healthy and asymptomatic throughout follow‐up. Although liver‐function or inflammatory markers were not collected, there was no clinical evidence of systemic inflammation or hepatic impairment. Despite the small sample size, these findings are consistent with the expected degradation and resorption profile of cross‐linked HA and align with known HA degradation and lymphatic clearance mechanisms [33]. These data provide preliminary insights into sHA kinetics following high‐volume injection of crosslinked HA filler. To our knowledge, no previous clinical studies have assessed serum HA behavior following dermal filler injection in humans, underscoring the novelty and limitations of this approach. Preclinical data support the plausibility of these findings. Reed et al. demonstrated in rabbits that subcutaneously injected [^3^H]‐hyaluronan is rapidly mobilized into the circulation and cleared primarily via lymphatic pathways, with a half‐life of approximately 16 h for the free pool [34].

Limitations of this study include a limited sample size, absence of a control group, and the pilot nature of serum analysis. Specifically, the serum HA substudy was limited to only three participants with follow‐up restricted to 90 days, which precludes conclusions about long‐term systemic kinetics. In addition to the different volumes used per patient, as the study was conducted in two centers with multiple investigators, while standardized protocols were applied to minimize inter‐operator variability, minor differences in injection technique cannot be fully excluded. Despite these constraints, the multimodal evaluation combining imaging, patient‐reported outcomes, and biochemical analysis strengthens the preliminary evidence presented here.

## 5 Conclusion

This prospective study provides evidence that HA filler is safe and effective for non‐surgical gluteal augmentation and contouring. Clinical assessments demonstrated consistent volumization, sustained outcomes, and high patient satisfaction throughout 12‐month follow‐up. These findings corroborate the growing body of literature, indicating that HA fillers can serve as minimally invasive alternatives for body contouring, particularly in anatomically demanding areas such as the gluteal region.

## Author Contributions

Study design and supervision: Gladstone Faria, Maria Bussade, Renata Viana, Ricardo Boggio. Clinical procedures and data collection: Gladstone Faria, Marta Barros, Caroline Mourão, Melina Kichler, Renata Viana, Luciana Zattar. Data analysis and interpretation: Renata Viana, Luciana Zattar. Manuscript drafting and revision: All authors.

## Ethics Statement

This study was conducted in accordance with the Declaration of Helsinki and Good Clinical Practice (GCP) guidelines. The protocol was approved by the institutional ethics committee (approval number 6.003.569).

## Consent

All participants provided written informed consent, including authorization for the use of images and data for publication.

## Conflicts of Interest

This study was supported by Ilikia Brasil, which provided the hyaluronic acid filler used. Renata Viana serves as a scientific consultant for Ilikia. The authors declare no other potential conflicts of interest with respect to the research, authorship, or publication of this article.

## Data Availability

The datasets generated and/or analyzed during the current study are available from the corresponding author on reasonable request.
